# Supplementary material for: Public questions spur the discovery of new bacterial species associated with lignin bioconversion of industrial waste
Source: R Soc Open Sci. 2019 Mar 20;6(3):180748. doi: 10.1098/rsos.180748 (PMC6458430; doi:10.1098/rsos.180748)
Supplement: Methods and supplementary Tables [file rsos180748supp1.docx]

**Supplementary Materials**

**Supplementary Methods**

**Data Acquisition by LC/MS/MS**

LC/MS/MS analyses were performed using an Easy nLC 1000 liquid chromatograph coupled to an Orbitrap Elite mass spectrometer (Thermo Scientific). Samples were injected onto a PepMap 100 C18, 5 µm, trapping column and separated by in-line gradient elution onto a 75 µm id x 20 cm PicoFrit capillary (New Objective) packed in-house with 3 µm “Magic” C18 stationary phase (Bruker-Michrom). The mobile phases consisted of (A) 0.1% formic acid/2% acetonitrile in water and (B) 0.1% formic acid in acetonitrile. Peptide separation was performed using a 90 min gradient of 5%-40% Mobile Phase B at a flow rate of 300 nl/min. Data-dependent acquisition implementing dynamic exclusion was used to select the five most intense precursors for HCD fragmentation using typical instrument settings.

**LC/MS/MS Data Post-Acquisition Analysis**

Raw data files from the LC/MS/MS acquisitions were processed using Proteome Discoverer 1.4 (Thermo Scientific). Proteins were identified by searching against a *C. lapagei* protein sequence database with the Mascot 2.5 search engine (Matrix Science) using a precursor ion tolerance of 10 ppm and a product ion mass tolerance of 0.01 Da. These data were imported into Scaffold 4 (Proteome Software) where a protein identification was defined as having at least two unique peptides at an LFDR threshold of 0.1%.

**Supplementary Tables**

**Supplementary Table 1.** *C. lapagei* proteins identified after growth on cellulose, hemicellulose and lignin.

| **Cellulose** | **Hemicellulose** | **Lignin** | **Gene Number** | **Predicted Location** | **Function** |
| --- | --- | --- | --- | --- | --- |
| **Protein Metabolism and Amino Acid Modification** | | | | | |
| X | X | X | 1096 | Cytoplasmic | Aspartate ammonia-lyase (EC 4.3.1.1) |
| X |  |  | 1630 | Cytoplasmic | Branched-chain amino acid aminotransferase (EC 2.6.1.42) |
| X |  |  | 211 | Cytoplasmic | Urocanate hydratase (EC 4.2.1.49) |
| X |  | X | 691 | Cytoplasmic | 2,3,4,5-tetrahydropyridine-2,6-dicarboxylate N-succinyltransferase (EC 2.3.1.117) |
| X |  | X | 38 | Cytoplasmic | Aspartate aminotransferase (EC 2.6.1.1) |
| X |  | X | 1039 | Cytoplasmic | FKBP-type peptidyl-prolyl cis-trans isomerase FklB (EC 5.2.1.8) |
| X | X | X | 856 | Cytoplasmic | Threonine synthase (EC 4.2.3.1) |
| X |  |  | 860 | Cytoplasmic | Aerobic respiration control protein arcA |
| X |  |  | 633 | Cytoplasmic | Aminoacyl-histidine dipeptidase (Peptidase D) (EC 3.4.13.3) |
| X |  |  | 969 | Cytoplasmic | Arginine deiminase (EC 3.5.3.6) |
| X |  |  | 970 | Cytoplasmic | Carbamate kinase (EC 2.7.2.2) |
| X |  |  | 701 | Cytoplasmic | Glutamate-1-semialdehyde aminotransferase (EC 5.4.3.8) |
| X |  |  | 961 | Cytoplasmic | Ornithine carbamoyltransferase (EC 2.1.3.3) |
| X | X |  | 971 | Cytoplasmic | Ornithine carbamoyltransferase (EC 2.1.3.3) |
| X |  |  | 60 | Cytoplasmic | Phosphoserine aminotransferase (EC 2.6.1.52) |
| X |  |  | 916 | Cytoplasmic | Succinate-semialdehyde dehydrogenase [NAD] (EC 1.2.1.24); Succinate-semialdehyde dehydrogenase [NAD(P)+] (EC 1.2.1.16) |
| X | X | X | 4114 | Cytoplasmic | Flavoprotein WrbA |
| X |  |  | 2004 | Cytoplasmic | Cytosol aminopeptidase PepA (EC 3.4.11.1) |
| X | X | X | 1593 | Cytoplasmic | ATP-dependent protease HslV (EC 3.4.25.-) |
| X |  |  | 1592 | Cytoplasmic | ATP-dependent hsl protease ATP-binding subunit HslU |
| X | X | X | 2771 | Cytoplasmic | 3-oxoacyl-[acyl-carrier-protein] synthase, KASI (EC 2.3.1.41) |
| X | X | X | 2347 | Cytoplasmic | Glycine cleavage system H protein |
| X |  |  | 2348 | Cytoplasmic | Glycine dehydrogenase [decarboxylating] (glycine cleavage system P protein) (EC 1.4.4.2) |
| X |  |  | 1862 | Cytoplasmic | Peptidyl-prolyl cis-trans isomerase PpiA precursor (EC 5.2.1.8) |
| X | X | X | 844 | Cytoplasmic | Chaperone protein DnaK |
| X |  | X | 464 | Cytoplasmic | Chaperone protein HtpG |
| X | X | X | 2605 | Cytoplasmic | ClpB protein |
| X | X | X | 1092 | Cytoplasmic | Heat shock protein 60 family chaperone GroEL |
| X | X | X | 1093 | Cytoplasmic | Heat shock protein 60 family co-chaperone GroES |
| X | X | X | 2583 | Cytoplasmic | Heat shock protein GrpE |
| X | X | X | 1530 | Cytoplasmic | Periplasmic thiol:disulfide interchange protein DsbA |
| X |  |  | 1470 | Cytoplasmic | 16 kDa heat shock protein B |
| X |  |  | 1838 | Cytoplasmic | 33 kDa chaperonin (Heat shock protein 33) (HSP33) |
| X |  |  | 514 | Cytoplasmic | ATP-dependent Clp protease ATP-binding subunit Clpx |
| X |  |  | 515 | Cytoplasmic | ATP-dependent Clp protease proteolytic subunit (EC 3.4.21.92) |
| X |  |  | 513 | Cytoplasmic | ATP-dependent protease La (EC 3.4.21.53) Type I |
| X | X |  | 1888 | Cytoplasmic | FKBP-type peptidyl-prolyl cis-trans isomerase FkpA precursor (EC 5.2.1.8) |
| X |  |  | 1886 | Cytoplasmic | FKBP-type peptidyl-prolyl cis-trans isomerase SlyD (EC 5.2.1.8) |
| X |  |  | 694 | Cytoplasmic | HtrA protease/chaperone protein |
| X |  |  | 435 | Cytoplasmic | Peptidyl-prolyl cis-trans isomerase PpiB (EC 5.2.1.8) |
| X |  |  | 1374 | Cytoplasmic | Protein export cytoplasm chaperone protein (SecB, maintains protein to be exported in unfolded state) |
| X |  |  | 3920 | Cytoplasmic | Ribulosamine/erythrulosamine 3-kinase potentially involved in protein deglycation |
| **Virulence Disease and Defense** | | | | | |
| X |  |  | 2067 | Cytoplasmic | 21 kDa hemolysin precursor |
| X |  |  | 556 | Cytoplasmic | FIG002283: Isochorismatase family protein |
| X |  |  | 2856 | Cytoplasmic | Proteinase inhibitor I11, ecotin precursor |
| X |  |  | 3970 | Cytoplasmic | Serine protein kinase (prkA protein), P-loop containing |
| X |  |  | 2586 | Cytoplasmic | Signal recognition particle, subunit Ffh SRP54 (TC 3.A.5.1.1) |
| X |  |  | 1746 | Cytoplasmic | Polymyxin resistance protein ArnC, glycosyl transferase (EC 2.4.-.-) |
| X | X | X | 3612 | Cytoplasmic | Acetolactate synthase, catabolic (EC 2.2.1.6) |
| X | X | X | 2731 | Cytoplasmic | Phosphotransferase system, phosphocarrier protein HPr |
| X |  |  | 3015 | Cytoplasmic | dTDP-glucose 4,6-dehydratase (EC 4.2.1.46) |
|  |  | X | 2270 | Cytoplasmic | Rhs-family protein |
| **Nucleotides and Nucleosides** | | | | | |
| X | X | X | 1505 | Cytoplasmic | ATP synthase alpha chain (EC 3.6.3.14) |
| X |  | X | 1503 | Cytoplasmic | ATP synthase beta chain (EC 3.6.3.14) |
| X |  |  | 686 | Cytoplasmic | Uridine monophosphate kinase (EC 2.7.4.22) |
| X |  |  | 1506 | Cytoplasmic | ATP synthase delta chain (EC 3.6.3.14) |
| X |  |  | 1502 | Cytoplasmic | ATP synthase epsilon chain (EC 3.6.3.14) |
| X | X |  | 1507 | Cytoplasmic | ATP synthase F0 sector subunit b (EC 3.6.3.14) |
| X | X |  | 1504 | Cytoplasmic | ATP synthase gamma chain (EC 3.6.3.14) |
| **Biosynthesis** | | | | | |
|  | X | X | 1873 | Cytoplasmic | Malonate decarboxylase alpha subunit |
| X |  | X | 463 | Cytoplasmic | Adenylate kinase (EC 2.7.4.3) |
| X | X | X | 976 | Cytoplasmic | Bona fide RidA/YjgF/TdcF/RutC subgroup |
| X |  | X | 1061 | Cytoplasmic | Adenylosuccinate synthetase (EC 6.3.4.4) |
| X |  |  | 1958 | Cytoplasmic | Biotin carboxyl carrier protein of acetyl-CoA carboxylase |
| X |  |  | 1957 | Cytoplasmic | Biotin carboxylase of acetyl-CoA carboxylase (EC 6.3.4.14) |
| X |  |  | 4076 | Cytoplasmic | Glucans biosynthesis protein G precursor |
| X |  |  | 672 | Cytoplasmic | Acetyl-coenzyme A carboxyl transferase alpha chain (EC 6.4.1.2) |
|  | X |  | 3102 | Cytoplasmic | Long-chain-fatty-acid--CoA ligase (EC 6.2.1.3) |
| X |  |  | 541 | Cytoplasmic | 6,7-dimethyl-8-ribityllumazine synthase (EC 2.5.1.78) |
| X |  |  | 3171 | Cytoplasmic | Dihydroneopterin triphosphate pyrophosphohydolase type 2 |
| X |  |  | 2326 | Cytoplasmic | Biosynthetic arginine decarboxylase (EC 4.1.1.19) |
|  | X |  | 2313 | Cytoplasmic | Nucleoside 5-triphosphatase RdgB (dHAPTP, dITP, xTP-specific) (EC 3.6.1.15) |
| X |  |  | 832 | Cytoplasmic | Carbamoyl-phosphate synthase large chain (EC 6.3.5.5) |
| X |  |  | 879 | Cytoplasmic | Phosphopentomutase (EC 5.4.2.7) |
| X |  |  | 878 | Cytoplasmic | Purine nucleoside phosphorylase (EC 2.4.2.1) |
| X |  |  | 880 | Cytoplasmic | Thymidine phosphorylase (EC 2.4.2.4) |
| X |  |  | 1024 | Cytoplasmic | 2',3'-cyclic-nucleotide 2'-phosphodiesterase (EC 3.1.4.16) |
|  | X |  | 1411 | Cytoplasmic | GTP pyrophosphokinase (EC 2.7.6.5), (p)ppGpp synthetase II / Guanosine-3',5'-bis(diphosphate) 3'-pyrophosphohydrolase (EC 3.1.7.2) |
| X | X | X | 4031 | Cytoplasmic | Acyl carrier protein |
|  | X |  | 2514 | Cytoplasmic | Carbon storage regulator |
| **Capsule** | | | | | |
|  | X |  | 1705 | Cytoplasmic | Cellulose synthase catalytic subunit [UDP-forming] (EC 2.4.1.12) |
|  | X |  | 2992 | Cytoplasmic | Colanic acid biosysnthesis glycosyl transferase WcaI |
| **Cell Division** | | | | | |
| X |  | X | 73 | Cytoplasmic | Cell division protein FtsK |
| X |  | X | 516 | Cytoplasmic | Cell division trigger factor (EC 5.2.1.8) |
|  |  | X | 42 | Cytoplasmic | Chromosome partition protein MukB |
| X |  |  | 2041 | Cytoplasmic | Cell division protein FtsH (EC 3.4.24.-) |
| X |  |  | 3235 | Cytoplasmic | Cell division topological specificity factor MinE |
| X |  |  | 1964 | Cytoplasmic | Rod shape-determining protein MreB |
| X |  |  | 3236 | Cytoplasmic | Septum site-determining protein MinD |
| **Motility** | | | | | |
|  | X | X | 3091 | Cytoplasmic | Flagellar biosynthesis protein FliC |
|  | X | X | 3092 | Cytoplasmic | Flagellar biosynthesis protein FliC |
| Membrane | | | | | |
| X | X | X | 235 | Cytoplasmic | 18K peptidoglycan-associated outer membrane lipoprotein; Peptidoglycan-associated lipoprotein precursor; Outer membrane protein P6; OmpA/MotB precursor |
| X |  | X | 3873 | Cytoplasmic | major outer membrane lipoprotein |
| X | X | X | 4137 | Cytoplasmic | Outer membrane protein A precursor |
| X |  |  | 526 | Cytoplasmic | FIG001943: hypothetical protein YajQ |
| X |  |  | 1063 | Cytoplasmic | HflC protein |
| X |  |  | 1064 | Cytoplasmic | HflK protein |
| X |  |  | 518 | Cytoplasmic | Hypothetical lipoprotein YajG precursor |
| X |  |  | 544 | Cytoplasmic | Hypothetical lipoprotein yajI |
| X |  |  | 1928 | Cytoplasmic | Large-conductance mechanosensitive channel |
| X |  |  | 2701 | Cytoplasmic | Outer membrane protein NlpB, lipoprotein component of the protein assembly complex (forms a complex with YaeT, YfiO, and YfgL); Lipoprotein-34 precursor |
|  | X |  | 3828 | Cytoplasmic | Phosphoethanolamine transferase EptA specific for the 1 phosphate group of core-lipid A |
| X |  |  | 2108 | Cytoplasmic | Uncharacterized membrane protein YqjD |
| X | X |  | 72 | Periplasmic | Outer membrane lipoprotein carrier protein LolA |
| **Metabolism** | | | | | |
| X | X | X | 2732 | Cytoplasmic | Cysteine synthase (EC 2.5.1.47) |
| X |  | X | 1604 | Cytoplasmic | Glycerol dehydrogenase (EC 1.1.1.6) |
| X |  | X | 1587 | Cytoplasmic | Glycerol kinase (EC 2.7.1.30) |
| X | X | X | 1981 | Cytoplasmic | Malate dehydrogenase (EC 1.1.1.37) |
| X |  | X | 3894 | Cytoplasmic | Phosphoenolpyruvate synthase (EC 2.7.9.2) |
| X | X | X | 2331 | Cytoplasmic | Phosphoglycerate kinase (EC 2.7.2.3) |
| X |  | X | 2329 | Cytoplasmic | Transketolase (EC 2.2.1.1) |
| X | X | X | 2714 | Cytoplasmic | Transketolase (EC 2.2.1.1) |
| X |  |  | 3319 | Cytoplasmic | 2-Keto-3-deoxy-D-manno-octulosonate-8-phosphate synthase (EC 2.5.1.55) |
| X |  |  | 2099 | Cytoplasmic | 2-ketobutyrate formate-lyase (EC 2.3.1.-) / Pyruvate formate-lyase (EC 2.3.1.54) |
| X |  |  | 3013 | Cytoplasmic | 6-phosphogluconate dehydrogenase, decarboxylating (EC 1.1.1.44) |
| X |  |  | 2803 | Cytoplasmic | Acetate kinase (EC 2.7.2.1) |
| X |  |  | 1658 | Cytoplasmic | Adenylate cyclase (EC 4.6.1.1) |
| X |  |  | 3994 | Cytoplasmic | Adenylosuccinate lyase (EC 4.3.2.2) |
| X |  |  | 1383 | Cytoplasmic | ADP-L-glycero-D-manno-heptose-6-epimerase (EC 5.1.3.20) |
| X | X |  | 1976 | Cytoplasmic | Aldehyde dehydrogenase B (EC 1.2.1.22) |
| X |  |  | 2346 | Cytoplasmic | Aminomethyltransferase (glycine cleavage system T protein) (EC 2.1.2.10) |
| X |  |  | 1812 | Cytoplasmic | Aspartate-semialdehyde dehydrogenase (EC 1.2.1.11) |
| X |  |  | 3855 | Cytoplasmic | Carbonic anhydrase (EC 4.2.1.1) |
| X |  |  | 2444 | Cytoplasmic | CTP synthase (EC 6.3.4.2) |
| X | X |  | 4092 | Cytoplasmic | D-3-phosphoglycerate dehydrogenase (EC 1.1.1.95) |
| X |  |  | 3108 | Cytoplasmic | D-cysteine desulfhydrase (EC 4.4.1.15) |
| X |  |  | 2061 | Cytoplasmic | FIG002208: Acetyltransferase (EC 2.3.1.-) |
| X | X |  | 1499 | Cytoplasmic | Glucosamine--fructose-6-phosphate aminotransferase [isomerizing] (EC 2.6.1.16) |
| X |  |  | 3184 | Cytoplasmic | Glucose-6-phosphate 1-dehydrogenase (EC 1.1.1.49) |
| X |  |  | 3813 | Cytoplasmic | Glutathione S-transferase (EC 2.5.1.18) |
| X |  |  | 2320 | Cytoplasmic | Glutathione synthetase (EC 6.3.2.3) |
| X |  |  | 1816 | Cytoplasmic | Glycogen synthase, ADP-glucose transglucosylase (EC 2.4.1.21) |
| X |  |  | 3831 | Cytoplasmic | Lactoylglutathione lyase (EC 4.4.1.5) |
| X |  |  | 3793 | Cytoplasmic | Mannose-6-phosphate isomerase (EC 5.3.1.8) |
| X |  |  | 3929 | Cytoplasmic | N,N'-diacetylchitobiose-specific 6-phospho-beta-glucosidase (EC 3.2.1.86) |
| X |  |  | 3534 | Cytoplasmic | NAD-dependent malic enzyme (EC 1.1.1.38) |
| X |  |  | 2716 | Cytoplasmic | NADP-dependent malic enzyme (EC 1.1.1.40) |
| X | X |  | 1734 | Cytoplasmic | Oligopeptidase A (EC 3.4.24.70) |
| X | X |  | 2802 | Cytoplasmic | Phosphate acetyltransferase (EC 2.3.1.8) |
| X |  |  | 3012 | Cytoplasmic | Phosphomannomutase (EC 5.4.2.8) |
| X |  |  | 2053 | Cytoplasmic | Polyribonucleotide nucleotidyltransferase (EC 2.7.7.8) |
| X |  |  | 1682 | Cytoplasmic | Putative carboxymethylenebutenolidase (EC 3.1.1.45) |
| X |  |  | 4040 | Cytoplasmic | Ribonuclease E (EC 3.1.26.12) |
| X |  |  | 2338 | Cytoplasmic | Ribose 5-phosphate isomerase A (EC 5.3.1.6) |
| X |  |  | 2325 | Cytoplasmic | S-adenosylmethionine synthetase (EC 2.5.1.6) |
| X |  |  | 2715 | Cytoplasmic | Transaldolase (EC 2.2.1.2) |
| X | X |  | 3253 | Cytoplasmic | Trehalase (EC 3.2.1.28); Periplasmic trehalase precursor (EC 3.2.1.28) |
| X |  |  | 2027 | Cytoplasmic | UDP-N-acetylglucosamine 1-carboxyvinyltransferase (EC 2.5.1.7) |
| X |  |  | 2688 | Cytoplasmic | Uracil phosphoribosyltransferase (EC 2.4.2.9) |
| X |  |  | 1683 | Cytoplasmic | Uridine phosphorylase (EC 2.4.2.3) |
| X |  |  | 3000 | Cytoplasmic | UTP--glucose-1-phosphate uridylyltransferase (EC 2.7.7.9) |
| X |  |  | 3338 | Cytoplasmic | UTP--glucose-1-phosphate uridylyltransferase (EC 2.7.7.9) |
| X |  |  | 3967 | Cytoplasmic | Aldose 1-epimerase family protein YeaD |
| X |  |  | 126 | Cytoplasmic Membrane | D-alanyl-D-alanine carboxypeptidase (EC 3.4.16.4) |
| X | X |  | 496 | Periplasmic | Glycoprotein-polysaccharide metabolism |
| X |  | X | 1382 | Cytoplasmic | 2-amino-3-ketobutyrate coenzyme A ligase (EC 2.3.1.29) |
| X |  |  | 2678 | Cytoplasmic | GMP synthase [glutamine-hydrolyzing] (EC 6.3.5.2) |
| X | X |  | 2784 | Cytoplasmic | Lysine-arginine-ornithine-binding periplasmic protein precursor (TC 3.A.1.3.1) |
| X |  |  | 3833 | Cytoplasmic | Probable monothiol glutaredoxin GrlA |
| X |  |  | 3642 | Cytoplasmic | Phenylacetic acid degradation protein PaaY |
| X |  |  | 1373 | Cytoplasmic | Glycerol-3-phosphate dehydrogenase [NAD(P)+] (EC 1.1.1.94) |
| X | X |  | 228 | Cytoplasmic | Phosphoglycerate mutase (EC 5.4.2.1) |
| X | X | X | 2332 | Cytoplasmic | Fructose-bisphosphate aldolase class II (EC 4.1.2.13) |
| X | X | X | 1578 | Cytoplasmic | 6-phosphofructokinase (EC 2.7.1.11) |
| X | X | X | 2445 | Cytoplasmic | Enolase (EC 4.2.1.11) |
| X |  | X | 2957 | Cytoplasmic | Fructose-bisphosphate aldolase class I (EC 4.1.2.13) |
| X |  | X | 1007 | Cytoplasmic | Inorganic pyrophosphatase (EC 3.6.1.1) |
|  | X | X | 3258 | Cytoplasmic | NAD-dependent glyceraldehyde-3-phosphate dehydrogenase (EC 1.2.1.12) |
| X | X | X | 3966 | Cytoplasmic | NAD-dependent glyceraldehyde-3-phosphate dehydrogenase (EC 1.2.1.12) |
| X |  | X | 3182 | Cytoplasmic | Pyruvate kinase (EC 2.7.1.40) |
| X |  | X | 1582 | Cytoplasmic | Triosephosphate isomerase (EC 5.3.1.1) |
| X |  |  | 1837 | Cytoplasmic | Phosphoenolpyruvate carboxykinase [ATP] (EC 4.1.1.49) |
| X |  |  | 1160 | Cytoplasmic | Acetyl-coenzyme A synthetase (EC 6.2.1.1) |
| X |  |  | 1000 | Cytoplasmic | Fructose-1,6-bisphosphatase, type I (EC 3.1.3.11) |
| X | X |  | 4116 | Cytoplasmic | Glucose-1-phosphatase (EC Glycolysis 3.1.3.10) |
| X |  |  | 1260 | Cytoplasmic | Glucose-6-phosphate isomerase (EC 5.3.1.9) |
| X |  |  | 3631 | Cytoplasmic | NAD-dependent glyceraldehyde-3-phosphate dehydrogenase (EC 1.2.1.12) |
| X |  |  | 2748 | Cytoplasmic | Pyruvate decarboxylase (EC 4.1.1.1); Alpha-keto-acid decarboxylase (EC 4.1.1.-) |
| X |  |  | 3872 | Cytoplasmic | Pyruvate kinase (EC 2.7.1.40) |
| X |  |  | 4130 | Cytoplasmic | Succinyl-CoA synthetase, alpha subunit-related enzymes |
| X |  |  | 3918 | Cytoplasmic | 6-phosphofructokinase class II (EC 2.7.1.11) |
| X |  | X | 1271 | Cytoplasmic | Isocitrate lyase (EC 4.1.3.1) |
| X |  |  | 1538 | Cytoplasmic | Glutamine synthetase type I (EC 6.3.1.2) |
| X |  |  | 1272 | Cytoplasmic | Malate synthase (EC 2.3.3.9) |
| X |  |  | 1365 | Cytoplasmic | Mannitol-1-phosphate 5-dehydrogenase (EC 1.1.1.17) |
| X |  |  | 3628 | Cytoplasmic | S-(hydroxymethyl)glutathione dehydrogenase (EC 1.1.1.284) |
| X |  |  | 3767 | Cytoplasmic | NAD(P) transhydrogenase subunit beta (EC 1.6.1.2) |
| X | X | X | 852 | Cytoplasmic | Transaldolase (EC 2.2.1.2) |
|  | X |  | 138 | Cytoplasmic | Transaldolase (EC 2.2.1.2) |
| X |  |  | 3299 | Cytoplasmic | Phenolic acid decarboxylase (EC 4.1.1.-) |
| X |  | X | 729 | Cytoplasmic | Aconitate hydratase 2 (EC 4.2.1.3) |
| X | X | X | 734 | Cytoplasmic | Dihydrolipoamide dehydrogenase (EC 1.8.1.4) / Dihydrolipoamide dehydrogenase of pyruvate dehydrogenase complex (EC 1.8.1.4) |
| X | X | X | 251 | Cytoplasmic | Dihydrolipoamide succinyltransferase component (E2) of 2-oxoglutarate dehydrogenase complex (EC 2.3.1.61) |
| X | X | X | 3989 | Cytoplasmic | Isocitrate dehydrogenase [NADP] (EC 1.1.1.42) |
| X |  | X | 736 | Cytoplasmic | Pyruvate dehydrogenase E1 component (EC 1.2.4.1) |
| X |  | X | 254 | Cytoplasmic | Succinate dehydrogenase flavoprotein subunit (EC 1.3.99.1) |
| X | X | X | 249 | Cytoplasmic | Succinyl-CoA ligase [ADP-forming] alpha chain (EC 6.2.1.5) |
| X | X | X | 250 | Cytoplasmic | Succinyl-CoA ligase [ADP-forming] beta chain (EC 6.2.1.5) |
| X |  |  | 252 | Cytoplasmic | 2-oxoglutarate dehydrogenase E1 component (EC 1.2.4.2) |
| X |  |  | 3384 | Cytoplasmic | Aconitate hydratase (EC 4.2.1.3) @ 2-methylisocitrate dehydratase (EC 4.2.1.99) |
| X |  |  | 257 | Cytoplasmic | Citrate synthase (si) (EC 2.3.3.1) |
| X |  |  | 735 | Cytoplasmic | Dihydrolipoamide acetyltransferase component of pyruvate dehydrogenase complex (EC 2.3.1.12) |
| X |  |  | 3791 | Cytoplasmic | Fumarate hydratase class II (EC 4.2.1.2) |
| X |  |  | 1079 | Cytoplasmic | Succinate dehydrogenase flavoprotein subunit (EC 1.3.99.1) |
| X |  | X | 3341 | Cytoplasmic | Alcohol dehydrogenase (EC 1.1.1.1); Acetaldehyde dehydrogenase (EC 1.2.1.10) |
| X |  | X | 64 | Cytoplasmic | Pyruvate formate-lyase (EC 2.3.1.54) |
| X |  |  | 2000 | Cytoplasmic | Alcohol dehydrogenase (EC 1.1.1.1) |
| X |  |  | 3531 | Cytoplasmic | Alcohol dehydrogenase (EC 1.1.1.1) |
| X |  |  | 1358 | Cytoplasmic | Aldehyde dehydrogenase B (EC 1.2.1.22) |
| X |  |  | 276 | Cytoplasmic | Phosphoglucomutase (EC 5.4.2.2) |
| X |  |  | 458 | Periplasmic | UDP-sugar hydrolase (EC 3.6.1.45); 5'-nucleotidase (EC 3.1.3.5) |
| **Metal** | | | | | |
| X | X |  | 3193 | Cytoplasmic | Copper resistance protein C precursor |
|  |  | X | 3516 | Cytoplasmic | Molybdopterin oxidoreductase (EC 1.2.1.2) @ selenocysteine-containing |
| X | X | X | 173 | Cytoplasmic | Non-specific DNA-binding protein Dps / Iron-binding ferritin-like antioxidant protein / Ferroxidase (EC 1.16.3.1) |
| X |  |  | 1898 | Cytoplasmic | Bacterioferritin |
| X |  |  | 280 | Cytoplasmic | Flavodoxin 1 |
| X |  |  | 281 | Cytoplasmic | Ferric uptake regulation protein FUR |
| X | X |  | 1572 | Cytoplasmic | Manganese superoxide dismutase (EC 1.15.1.1) |
| X |  |  | 168 | Cytoplasmic | Mn-dependent transcriptional regulator MntR |
| X |  |  | 253 | Cytoplasmic | Succinate dehydrogenase iron-sulfur protein (EC 1.3.99.1) |
| X |  |  | 1574 | Cytoplasmic | Copper sensory histidine kinase CpxA |
| X |  |  | 1575 | Cytoplasmic | Copper-sensing two-component system response regulator CpxR |
|  |  | X | 220 | Unknown | Molybdenum ABC transporter, periplasmic molybdenum-binding protein ModA (TC 3.A.1.8.1) |
| **Stress Response** | | | | | |
| X | X | X | 3540 | Cytoplasmic | Osmotically inducible protein C |
| X |  | X | 1835 | Cytoplasmic | Two-component system response regulator OmpR |
| X |  |  | 1087 | Cytoplasmic | Entericidin B precursor |
| X | X |  | 3934 | Cytoplasmic | Osmotically inducible lipoprotein E precursor |
| X |  |  | 3938 | Cytoplasmic | Periplasmic protein related to spheroblast formation |
| X | X |  | 3215 | Cytoplasmic | Cold shock protein CspC |
| X | X |  | 2827 | Cytoplasmic | ElaB protein |
| X |  |  | 2064 | Cytoplasmic | General stress protein 18 |
| X | X |  | 3439 | Cytoplasmic | Protein yciF |
| X |  |  | 1736 | Cytoplasmic | Universal stress protein A |
| X |  |  | 3766 | Cytoplasmic | Universal stress protein E |
| X |  |  | 1988 | Cytoplasmic | Stringent starvation protein A |
| **Oxidase** | | | | | |
| X | X | X | 341 | Cytoplasmic | Alkyl hydroperoxide reductase protein C (EC 1.6.4.-) |
| X | X | X | 553 | Cytoplasmic | Alkyl hydroperoxide reductase subunit C-like protein |
| X |  | X | 244 | Cytoplasmic | Cytochrome d ubiquinol oxidase subunit I (EC 1.10.3.-) |
| X |  | X | 3399 | Cytoplasmic | Enoyl-[acyl-carrier-protein] reductase [NADH] (EC 1.3.1.9) |
| X |  | X | 2842 | Cytoplasmic | Ribonucleotide reductase of class Ia (aerobic), alpha subunit (EC 1.17.4.1) |
| X |  | X | 3426 | Cytoplasmic | Thiol peroxidase, Tpx-type (EC 1.11.1.15) |
| X | X | X | 904 | Cytoplasmic | Organic hydroperoxide resistance protein |
| X | X | X | 1639 | Cytoplasmic | Thioredoxin |
| X | X |  | 4032 | Cytoplasmic | 3-oxoacyl-[acyl-carrier protein] reductase (EC 1.1.1.100) |
| X |  |  | 3673 | Cytoplasmic | Aldo-keto reductase |
| X | X |  | 2695 | Cytoplasmic | Arsenate reductase (EC 1.20.4.1) |
| X |  |  | 1603 | Cytoplasmic | Catalase (EC 1.11.1.6) / Peroxidase (EC 1.11.1.7) |
| X |  |  | 243 | Cytoplasmic | Cytochrome d ubiquinol oxidase subunit II (EC 1.10.3.-) |
| X |  |  | 521 | Cytoplasmic | Cytochrome O ubiquinol oxidase subunit I (EC 1.10.3.-) |
| X |  |  | 1732 | Cytoplasmic | Glutathione reductase (EC 1.8.1.7) |
| X |  |  | 2226 | Cytoplasmic | Methylglyoxal reductase, acetol producing (EC 1.1.1.-) / 2,5-diketo-D-gluconate reductase A (EC 1.1.1.274) |
| X |  |  | 2813 | Cytoplasmic | NADH-ubiquinone oxidoreductase chain C (EC 1.6.5.3) / NADH-ubiquinone oxidoreductase chain D (EC 1.6.5.3) |
| X |  |  | 2815 | Cytoplasmic | NADH-ubiquinone oxidoreductase chain F (EC 1.6.5.3) |
| X | X |  | 1030 | Cytoplasmic | NADPH:quinone oxidoreductase 2 |
| X |  |  | 3826 | Cytoplasmic | Putative aldo/keto reductase |
| X |  |  | 3467 | Cytoplasmic | Putative oxidoreductase YncB |
| X |  |  | 92 | Cytoplasmic | Pyruvate oxidase [ubiquinone, cytochrome] (EC 1.2.2.2) |
| X |  |  | 1747 | Cytoplasmic | UDP-glucuronic acid oxidase (UDP-4-keto-hexauronic acid decarboxylating) (EC 1.1.1.305) / UDP-4-amino-4-deoxy-L-arabinose formyltransferase (EC 2.1.2.13) |
| X |  |  | 75 | Signal Peptide, Unknown | Thioredoxin reductase (EC 1.8.1.9) |
| X |  |  | 2408 | Cytoplasmic | Prepilin peptidase dependent protein A precursor |
| **Phage** | | | | | |
| X |  | X | 1774 | Cytoplasmic | DcrB protein precursor |
| X | X | X | 3909 | Cytoplasmic | Integration host factor alpha subunit |
| X |  |  | 34 | Cytoplasmic | Integrase |
|  | X |  | 2142 | Cytoplasmic | Phage protein |
| X | X |  | 3406 | Cytoplasmic | Phage shock protein A |
|  | X |  | 2159 | Cytoplasmic | Phage tail protein # FIG072132 and FIG003673 |
| **Quorum Sensing** | | | | | |
| X | X | X | 637 | Cytoplasmic | Phosphoheptose isomerase 1 (EC 5.3.1.-) |
| X |  |  | 2129 | Cytoplasmic | Autoinducer 2 (AI-2) aldolase LsrF (EC 4.2.1.-) |
| X |  |  | 2518 | Cytoplasmic | S-ribosylhomocysteine lyase (EC 4.4.1.21) / Autoinducer-2 production protein LuxS |
| **Reactive Oxygen** | | | | | |
|  | X | X | 4142 | Cytoplasmic | Paraquat-inducible protein B |
| X | X | X | 3835 | Cytoplasmic | Superoxide dismutase [Fe] (EC 1.15.1.1) |
| X |  |  | 2354 | Cytoplasmic | Folate-dependent protein for Fe/S cluster synthesis/repair in oxidative stress |
| X |  |  | 2359 | Cytoplasmic | Thiol:disulfide interchange protein DsbC |
| X |  |  | 1655 | Cytoplasmic | Homolog of E. coli Hemx protein |
| **Replication, Transcription, Translation** | | | | | |
|  |  | X | 310 | Unknown | DNA polymerase III delta subunit (EC 2.7.7.7) |
| X |  | X | 1277 | Cytoplasmic | DNA-binding protein HU-alpha |
| X |  | X | 865 | Cytoplasmic | Right origin-binding protein |
| X |  |  | 4133 | Cytoplasmic | DNA helicase IV |
| X |  |  | 2511 | Cytoplasmic | RecA protein |
| X |  |  | 2879 | Cytoplasmic | Translation elongation factor P-related protein |
|  | X |  | 1977 | Cytoplasmic | probable ribonuclease inhibitor YPO3690 |
| X |  |  | 1980 | Cytoplasmic | Arginine pathway regulatory protein ArgR, repressor of arg regulon |
| X |  |  | 2846 | Cytoplasmic | DNA-binding capsular synthesis response regulator RcsB |
| X | X |  | 3339 | Cytoplasmic | DNA-binding protein H-NS |
| X |  |  | 3308 | Cytoplasmic | GTP-binding and nucleic acid-binding protein YchF |
| X |  |  | 1229 | Cytoplasmic | Single-stranded DNA-binding protein |
| X | X | X | 1925 | Cytoplasmic | DNA-directed RNA polymerase alpha subunit (EC 2.7.7.6) |
| X |  | X | 1292 | Cytoplasmic | DNA-directed RNA polymerase beta' subunit (EC 2.7.7.6) |
| X | X | X | 1295 | Cytoplasmic | LSU ribosomal protein L10p (P0) |
| X |  | X | 1297 | Cytoplasmic | LSU ribosomal protein L11p (L12e) |
| X |  | X | 1911 | Cytoplasmic | LSU ribosomal protein L14p (L23e) |
| X |  | X | 1926 | Cytoplasmic | LSU ribosomal protein L17p |
| X | X | X | 1296 | Cytoplasmic | LSU ribosomal protein L1p (L10Ae) |
| X |  | X | 3912 | Cytoplasmic | LSU ribosomal protein L20p |
| X |  | X | 1904 | Cytoplasmic | LSU ribosomal protein L2p (L8e) |
| X |  | X | 1901 | Cytoplasmic | LSU ribosomal protein L3p (L3e) |
| X |  | X | 1902 | Cytoplasmic | LSU ribosomal protein L4p (L1e) |
| X |  | X | 1916 | Cytoplasmic | LSU ribosomal protein L6p (L9e) |
| X | X | X | 1294 | Cytoplasmic | LSU ribosomal protein L7/L12 (P1/P2) |
|  |  | X | 1727 | Cytoplasmic | LysR family transcriptional regulator YhjC |
| X |  | X | 2362 | Cytoplasmic | Lysyl-tRNA synthetase (class II) (EC 6.1.1.6) |
| X | X | X | 3311 | Cytoplasmic | Ribose-phosphate pyrophosphokinase (EC 2.7.6.1) |
| X | X | X | 56 | Cytoplasmic | SSU ribosomal protein S1p |
| X |  | X | 1924 | Cytoplasmic | SSU ribosomal protein S4p (S9e) |
| X | X | X | 1918 | Cytoplasmic | SSU ribosomal protein S5p (S2e) |
| X |  | X | 1042 | Cytoplasmic | SSU ribosomal protein S6p |
| X |  | X | 1894 | Cytoplasmic | SSU ribosomal protein S7p (S5e) |
| X |  | X | 1915 | Cytoplasmic | SSU ribosomal protein S8p (S15Ae) |
| X |  | X | 1987 | Cytoplasmic | SSU ribosomal protein S9p (S16e) |
|  |  | X | 3915 | Cytoplasmic | Threonyl-tRNA synthetase (EC 6.1.1.3) |
| X | X | X | 1640 | Cytoplasmic | Transcription termination factor Rho |
| X |  | X | 4009 | Cytoplasmic | Transcription-repair coupling factor |
| X |  |  | 1059 | Cytoplasmic | 3'-to-5' exoribonuclease RNase R |
| X |  |  | 2782 | Cytoplasmic | Amidophosphoribosyltransferase (EC 2.4.2.14) |
| X |  |  | 36 | Cytoplasmic | Asparaginyl-tRNA synthetase (EC 6.1.1.22) |
| X |  |  | 881 | Cytoplasmic | Deoxyribose-phosphate aldolase (EC 4.1.2.4) |
| X | X |  | 1293 | Cytoplasmic | DNA-directed RNA polymerase beta subunit (EC 2.7.7.6) |
| X |  |  | 1808 | Cytoplasmic | Gluconate utilization system Gnt-I transcriptional repressor |
| X |  |  | 284 | Cytoplasmic | Glutaminyl-tRNA synthetase (EC 6.1.1.18) |
| X | X |  | 2740 | Cytoplasmic | Glutamyl-tRNA synthetase (EC 6.1.1.17) |
| X |  |  | 1330 | Cytoplasmic | Glycyl-tRNA synthetase alpha chain (EC 6.1.1.14) |
| X |  |  | 1329 | Cytoplasmic | Glycyl-tRNA synthetase beta chain (EC 6.1.1.14) |
| X |  |  | 838 | Cytoplasmic | Isoleucyl-tRNA synthetase (EC 6.1.1.5) |
| X |  |  | 1986 | Cytoplasmic | LSU ribosomal protein L13p (L13Ae) |
| X |  |  | 1920 | Cytoplasmic | LSU ribosomal protein L15p (L27Ae) |
| X |  |  | 1908 | Cytoplasmic | LSU ribosomal protein L16p (L10e) |
| X | X |  | 1917 | Cytoplasmic | LSU ribosomal protein L18p (L5e) |
| X |  |  | 2590 | Cytoplasmic | LSU ribosomal protein L19p |
| X |  |  | 2030 | Cytoplasmic | LSU ribosomal protein L21p |
| X |  |  | 1906 | Cytoplasmic | LSU ribosomal protein L22p (L17e) |
| X |  |  | 1912 | Cytoplasmic | LSU ribosomal protein L24p (L26e) |
| X |  |  | 1913 | Cytoplasmic | LSU ribosomal protein L5p (L11e) |
|  | X |  | 2102 | Cytoplasmic | LysR-family transcriptional regulator YhaJ |
| X |  |  | 3911 | Cytoplasmic | Phenylalanyl-tRNA synthetase alpha chain (EC 6.1.1.20) |
| X |  |  | 1275 | Cytoplasmic | Phosphoribosylamine--glycine ligase (EC 6.3.4.13) |
|  | X |  | 3019 | Cytoplasmic | Phosphoribosylformimino-5-aminoimidazole carboxamide ribotide isomerase (EC 5.3.1.16) |
| X |  |  | 753 | Cytoplasmic | Protein export cytoplasm protein SecA ATPase RNA helicase (TC 3.A.5.1.1) |
| X |  |  | 2601 | Cytoplasmic | Ribosome hibernation protein YfiA |
| X |  |  | 685 | Cytoplasmic | Ribosome recycling factor |
| X |  |  | 2175 | Cytoplasmic | RNA polymerase sigma factor RpoD |
| X |  |  | 2468 | Cytoplasmic | RNA polymerase sigma factor RpoS |
| X |  |  | 488 | Cytoplasmic | RNA signal recognition particle 4.5S RNA |
| X |  |  | 70 | Cytoplasmic | Seryl-tRNA synthetase (EC 6.1.1.11) |
| X | X |  | 1900 | Cytoplasmic | SSU ribosomal protein S10p (S20e) |
| X |  |  | 1893 | Cytoplasmic | SSU ribosomal protein S12p (S23e) |
| X |  |  | 1923 | Cytoplasmic | SSU ribosomal protein S13p (S18e) |
| X |  |  | 2587 | Cytoplasmic | SSU ribosomal protein S16p |
| X |  |  | 1040 | Cytoplasmic | SSU ribosomal protein S18p @ SSU ribosomal protein S18p, zinc-independent |
| X |  |  | 1905 | Cytoplasmic | SSU ribosomal protein S19p (S15e) |
| X |  |  | 688 | Cytoplasmic | SSU ribosomal protein S2p (SAe) |
| X |  |  | 1907 | Cytoplasmic | SSU ribosomal protein S3p (S3e) |
| X |  |  | 1298 | Cytoplasmic | Transcription antitermination protein NusG |
| X |  |  | 2047 | Cytoplasmic | Transcription termination protein NusA |
| X |  |  | 3210 | Cytoplasmic | Transcriptional regulator KdgR, KDG operon repressor |
| X |  |  | 3821 | Cytoplasmic | Transcriptional regulator SlyA |
| X |  |  | 932 | Cytoplasmic | Transcriptional regulator, GntR family domain / Aspartate aminotransferase (EC 2.6.1.1) |
| X |  |  | 1855 | Cytoplasmic | Tryptophanyl-tRNA synthetase (EC 6.1.1.2) |
| X |  |  | 2002 | Cytoplasmic | Valyl-tRNA synthetase (EC 6.1.1.9) |
| X |  |  | 1868 | Cytoplasmic | Cyclic AMP receptor protein |
| X |  |  | 339 | Cytoplasmic | Universal stress protein G |
| X | X | X | 1895 | Cytoplasmic | Translation elongation factor G |
| X |  | X | 1089 | Cytoplasmic | Translation elongation factor P |
| X | X | X | 687 | Cytoplasmic | Translation elongation factor Ts |
| X | X | X | 1896 | Cytoplasmic | Translation elongation factor Tu |
| X | X | X | 1300 | Cytoplasmic | Translation elongation factor Tu |
| X |  |  | 2048 | Cytoplasmic | Translation initiation factor 2 |
| X |  |  | 888 | Cytoplasmic | Peptide chain release factor 3 |
| X |  |  | 3488 | Cytoplasmic | Translation elongation factor G |
| X |  |  | 79 | Cytoplasmic | Translation initiation factor 1 |
| **Transport** | | | | | |
| X | X | X | 1307 | Cytoplasmic | Dipeptide-binding ABC transporter, periplasmic substrate-binding component (TC 3.A.1.5.2) |
| X | X | X | 2895 | Cytoplasmic | Galactose/methyl galactoside ABC transport system, D-galactose-binding periplasmic protein MglB (TC 3.A.1.2.3) |
| X |  | X | 74 | Cytoplasmic | Leucine-responsive regulatory protein, regulator for leucine (or lrp) regulon and high-affinity branched-chain amino acid transport system |
| X | X | X | 3343 | Cytoplasmic | Oligopeptide ABC transporter, periplasmic oligopeptide-binding protein OppA (TC 3.A.1.5.1) |
|  | X | X | 1494 | Cytoplasmic | Phosphate transport system regulatory protein PhoU |
| X | X | X | 2729 | Cytoplasmic | PTS system, glucose-specific IIA component (EC 2.7.1.69) |
|  |  | X | 3933 | Cytoplasmic | PTS system, N,N'-diacetylchitobiose-specific IIB component (EC 2.7.1.69) |
| X | X | X | 1522 | Cytoplasmic | Ribose ABC transport system, periplasmic ribose-binding protein RbsB (TC 3.A.1.2.1) |
| X | X | X | 2024 | Cytoplasmic | Uncharacterized ABC transporter, auxiliary component YrbC |
|  |  | X | 1077 | Cytoplasmic | Potassium efflux system KefA protein / Small-conductance mechanosensitive channel |
| X |  | X | 548 | Cytoplasmic | Protein-export membrane protein SecD (TC 3.A.5.1.1) |
| X |  |  | 870 | Cytoplasmic | ABC transporter, ATP-binding protein |
| X |  |  | 4002 | Cytoplasmic | ABC transporter, periplasmic spermidine putrescine-binding protein PotD (TC 3.A.1.11.1) |
| X | X |  | 2128 | Cytoplasmic | Autoinducer 2 (AI-2) ABC transport system, periplasmic AI-2 binding protein LsrB |
| X | X |  | 3107 | Cytoplasmic | Cystine ABC transporter, periplasmic cystine-binding protein FliY |
| X |  |  | 1788 | Cytoplasmic | High-affinity leucine-specific transport system, periplasmic binding protein LivK (TC 3.A.1.4.1) |
| X |  |  | 2785 | Cytoplasmic | Histidine ABC transporter, histidine-binding periplasmic protein precursor HisJ (TC 3.A.1.3.1) |
| X |  |  | 2015 | Cytoplasmic | Lipopolysaccharide ABC transporter, ATP-binding protein LptB |
| X |  |  | 2525 | Cytoplasmic | L-proline glycine betaine binding ABC transporter protein Prox (TC 3.A.1.12.1) |
| X |  |  | 1252 | Cytoplasmic | Maltose/maltodextrin ABC transporter, substrate binding periplasmic protein MalE |
| X |  |  | 660 | Cytoplasmic | Methionine ABC transporter substrate-binding protein |
| X | X |  | 2919 | Cytoplasmic | Osmoprotectant ABC transporter binding protein YehZ |
| X |  |  | 2730 | Cytoplasmic | Phosphoenolpyruvate-protein phosphotransferase of PTS system (EC 2.7.3.9) |
| X |  |  | 2012 | Cytoplasmic | PTS IIA-like nitrogen-regulatory protein PtsN |
| X |  |  | 2467 | Cytoplasmic | PTS system, maltose and glucose-specific IIC component (EC 2.7.1.69) / PTS system, maltose and glucose-specific IIB component (EC 2.7.1.69) |
| X |  |  | 1364 | Cytoplasmic | PTS system, mannitol-specific IIC component (EC 2.7.1.69) / PTS system, mannitol-specific IIB component (EC 2.7.1.69) / PTS system, mannitol-specific IIA component |
| X | X |  | 3222 | Cytoplasmic | PTS system, mannose-specific IIA component (EC 2.7.1.69) / PTS system, mannose-specific IIB component (EC 2.7.1.69) |
| X |  |  | 3220 | Cytoplasmic | PTS system, mannose-specific IID component |
| X | X |  | 1006 | Cytoplasmic | Putative sugar ABC transport system, periplasmic binding protein YtfQ precursor |
| X |  |  | 1945 | Cytoplasmic | Glutamate Aspartate periplasmic binding protein precursor GltI (TC 3.A.1.3.4) |
| X |  |  | 186 | Cytoplasmic | Predicted membrane fusion protein (MFP) component of efflux pump, membrane anchor protein YbhG |
|  |  | X | 472 | Cytoplasmic Membrane | Potassium efflux system KefA protein / Small-conductance mechanosensitive channel |
| X |  |  | 474 | Cytoplasmic Membrane | Membrane fusion protein of RND family multidrug efflux pump |
| X | X | X | 100 | Periplasmic | Arginine ABC transporter, periplasmic arginine-binding protein ArtI |
| X | X | X | 298 | Periplasmic | Glutamate Aspartate periplasmic binding protein precursor GltI (TC 3.A.1.3.4) |
| X | X |  | 174 | Periplasmic | Glutamine ABC transporter, periplasmic glutamine-binding protein (TC 3.A.1.3.2) |
| X |  |  | 111 | Periplasmic | Putrescine ABC transporter putrescine-binding protein PotF (TC 3.A.1.11.2) |
| **Unknown** | | | | | |
|  | X | X | 2254 | Cytoplasmic | FIG00642489: hypothetical protein |
|  | X | X | 1118 | Cytoplasmic | hypothetical protein |
| X | X | X | 887 | Cytoplasmic | Osmotically inducible protein OsmY |
| X |  | X | 692 | Cytoplasmic | UPF0325 protein YaeH |
| X |  |  | 303 | Cytoplasmic | FIG002095: hypothetical protein |
| X |  |  | 2805 | Cytoplasmic | FIG00361523: hypothetical protein |
| X |  |  | 1491 | Cytoplasmic | FIG00553873: hypothetical protein |
| X |  |  | 1806 | Cytoplasmic | FIG00553873: hypothetical protein |
| X |  |  | 2572 | Cytoplasmic | FIG00626262: hypothetical protein |
| X | X |  | 3470 | Cytoplasmic | FIG00626404: hypothetical protein |
| X |  |  | 2694 | Cytoplasmic | FIG00626489: hypothetical protein |
| X |  |  | 2533 | Cytoplasmic | FIG00626891: hypothetical protein |
| X |  |  | 3919 | Cytoplasmic | FIG00637864: hypothetical protein |
| X |  |  | 2758 | Cytoplasmic | FIG00637865: hypothetical protein |
| X |  |  | 2966 | Cytoplasmic | FIG00638559: hypothetical protein |
| X |  |  | 3297 | Cytoplasmic | FIG00638667: hypothetical protein |
| X | X |  | 4115 | Cytoplasmic | FIG00895798: hypothetical protein |
| X |  |  | 2216 | Cytoplasmic | FIG00896075: hypothetical protein |
| X |  |  | 3425 | Cytoplasmic | hypothetical protein |
|  | X |  | 626 | Cytoplasmic | InterPro IPR001950:IPR001993 |
| X | X |  | 2109 | Cytoplasmic | Periplasmic protein YqjC |
| X |  |  | 98 | Cytoplasmic | probable lipoprotein |
| X |  |  | 2423 | Cytoplasmic | probable lipoprotein |
| X | X |  | 1534 | Cytoplasmic | Protein of unknown function DUF414 |
| X |  |  | 3769 | Cytoplasmic | Protein ydgH precursor |
| X | X |  | 1022 | Cytoplasmic | Protein ytfJ precursor |
|  | X |  | 3068 | Cytoplasmic | putative cytoplasmic protein |
| X | X |  | 1829 | Cytoplasmic | Putative exported protein precursor |
|  | X |  | 3691 | Cytoplasmic | putative secreted protein |
| X |  |  | 1242 | Cytoplasmic | UPF0337 protein yjbJ |

**Supplementary Table 2. *C. lapagei* proteins also identified by ME Brown et al. in their extracellular proteome analysis of *Amycolatopsis* sp. 75iv2 grown on miscanthus lignocellulose.**

| **Cellulose** | **Hemicellulose** | **Lignin** | **Gene Number** | **Predicted Location** | **Function** |
| --- | --- | --- | --- | --- | --- |
| X |  |  | 60 | Cytoplasmic | Phosphoserine aminotransferase (EC 2.6.1.52) |
| X |  |  | 2348 | Cytoplasmic | Glycine dehydrogenase [decarboxylating] (glycine cleavage system P protein) (EC 1.4.4.2) |
| X | X | X | 1505 | Cytoplasmic | ATP synthase alpha chain (EC 3.6.3.14) |
| X | X | X | 2331 | Cytoplasmic | Phosphoglycerate kinase (EC 2.7.2.3) |
| X |  |  | 2715 | Cytoplasmic | Transaldolase (EC 2.2.1.2) |
| X |  |  | 3967 | Cytoplasmic | Aldose 1-epimerase family protein YeaD |
| X | X | X | 2332 | Cytoplasmic | Fructose-bisphosphate aldolase class II (EC 4.1.2.13) |
| X |  |  | 1837 | Cytoplasmic | Phosphoenolpyruvate carboxykinase [ATP] (EC 4.1.1.49) |
| X |  |  | 1260 | Cytoplasmic | Glucose-6-phosphate isomerase (EC 5.3.1.9) |
| X |  |  | 1538 | Cytoplasmic | Glutamine synthetase type I (EC 6.3.1.2) |
| X |  |  | 1365 | Cytoplasmic | Mannitol-1-phosphate 5-dehydrogenase (EC 1.1.1.17) |
| X | X | X | 852 | Cytoplasmic | Transaldolase (EC 2.2.1.2) |
| X |  | X | 729 | Cytoplasmic | Aconitate hydratase 2 (EC 4.2.1.3) |
| X | X | X | 734 | Cytoplasmic | Dihydrolipoamide dehydrogenase (EC 1.8.1.4) / Dihydrolipoamide dehydrogenase of pyruvate dehydrogenase complex (EC 1.8.1.4) |
| X | X | X | 251 | Cytoplasmic | Dihydrolipoamide succinyltransferase component (E2) of 2-oxoglutarate dehydrogenase complex (EC 2.3.1.61) |
| X | X | X | 3989 | Cytoplasmic | Isocitrate dehydrogenase [NADP] (EC 1.1.1.42) |
| X |  | X | 254 | Cytoplasmic | Succinate dehydrogenase flavoprotein subunit (EC 1.3.99.1) |
| X |  |  | 257 | Cytoplasmic | Citrate synthase (si) (EC 2.3.3.1) |
| X |  |  | 735 | Cytoplasmic | Dihydrolipoamide acetyltransferase component of pyruvate dehydrogenase complex (EC 2.3.1.12) |
| X |  | X | 3341 | Cytoplasmic | Alcohol dehydrogenase (EC 1.1.1.1); Acetaldehyde dehydrogenase (EC 1.2.1.10) |
| X |  |  | 1358 | Cytoplasmic | Aldehyde dehydrogenase B (EC 1.2.1.22) |
| X |  | X | 2842 | Cytoplasmic | Ribonucleotide reductase of class Ia (aerobic), alpha subunit (EC 1.17.4.1) |
| X |  | X | 3426 | Cytoplasmic | Thiol peroxidase, Tpx-type (EC 1.11.1.15) |
| X |  |  | 1603 | Cytoplasmic | Catalase (EC 1.11.1.6) / Peroxidase (EC 1.11.1.7) |
| X |  |  | 3467 | Cytoplasmic | Putative oxidoreductase YncB |
| X | X | X | 3311 | Cytoplasmic | Ribose-phosphate pyrophosphokinase (EC 2.7.6.1) |
